# *Flemingia macrophylla* Extract Ameliorates Experimental Osteoporosis in Ovariectomized Rats

**DOI:** 10.1093/ecam/nep179

**Published:** 2011-02-14

**Authors:** Hui-Ya Ho, Jin-Bin Wu, Wen-Chuan Lin

**Affiliations:** ^1^Graduate Institute of Pharmaceutical Chemistry, School of Pharmacy, China Medical University, Taichung, Taiwan; ^2^Department of Pharmacology, School of Medicine, Graduate Institute of Basic Medical Science and Tsuzuki Institute for Traditional Medicine, China Medical University, Taichung, Taiwan

## Abstract

*Flemingia macrophylla* (Leguminosae), a native plant of Taiwan, is used as folk medicine. An *in vitro* study showed that a 75% ethanolic extract of *F. macrophylla* (FME) inhibited osteoclast differentiation of cultured rat bone marrow cells, and the active component, lespedezaflavanone A (LDF-A), was isolated. It was found that oral administration of FME for 13 weeks suppressed bone loss in ovariectomized rats, an experimental model of osteoporosis. In addition, FME decreased urinary deoxypyridinoline concentrations but did not inhibit serum alkaline phosphatase activities, indicating that it ameliorated bone loss via inhibition of bone resorption. These results suggest that FME may represent a useful remedy for the treatment of bone resorption diseases, such as osteoporosis. In addition, LDF-A could be used as a marker compound to control the quality of FME.

## 1. Introduction

Osteoporosis is a major health problem in aging communities. Osteoporosis significantly increases the frequency fractures, and hip fracture in elderly patients is a very serious problem because it can limit the patient's quality of life. Bisphosphonates are currently the major drugs used to treat osteoporosis. However, they are associated with side effects as esophageal cancer and osteonecrosis of the jaw [1]. Therefore, it is still valuable to develop safer preventive medicine to suppress osteoporosis.

Osteoporosis is caused by an imbalance between bone resorption and bone formation, which results in bone loss and fractures after mineral flux [2]. Bisphosphonates are bone resorption inhibitors that maintain bone mass by inhibiting the function of osteoclasts [3]. Osteoclasts are the primary bone-resorbing cells and hematopoietic in origin, and their differentiation is induced by the local production of various cytokines, receptor activator of nuclear factor *κ*B ligand (RANKL), and macrophage colony-stimulating factor (M-CSF) [4, 5]. Thus, osteoclast formation in bone marrow cells may be applicable in the screening of osteoporosis medicines.

On the other hand, herbal medicine has been widely used for the treatment of rheumatism and bone disease. The root and stem of the herbal plant *Flemingia macrophylla* (Leguminosae) are recommended in Taiwan for the management of rheumatoid arthritis and bone disease, such as low-back pain in climacteric and senescent periods. Uncontrolled osteoclastogenesis is observed in rheumatoid arthritis, which is accompanied with significant bone resorption [6]. In cases of high-bone destruction, the function and differentiation of osteoclasts are highly upregulated. It has been suggested that the effectiveness of oriental medicines on low-back pain seems to be due to their efficacy in curing osteoporosis [7]. However, no data are available with regard to the recovery of bone mass by *F. macrophylla*.

In postmenopausal women, estrogen deficiency leads to the most common form of osteoporosis. Several medications have also been reported to be clinically effective in curing osteoporosis based on results obtained from nonsteroidal sources, such as bisphosphonates and calcitonin, using animal models [8]. Investigating postmenopausal osteoporosis using ovariectomized (OVX) rats is useful to evaluate osteoporosis drugs. In this study, we investigated the effects of FME on osteopenia in OVX rats.

## 2. Methods

### 2.1. Plant Extract Preparation

Roots and stems of *F. macrophylla* were purchased from a local market in Taichung, Taiwan. The plants were identified by the Institute of Chinese Pharmaceutical Sciences, China Medical University, where voucher specimens have been deposited.

*Flemingia macrophylla* roots and stems were extracted twice with 75% ethanol, followed by evaporation of the solvent under reduced pressure at 50°C. The ethanolic extract (FME) yield was 7.8%. The FME dosage used in the experiments was based on the dry weight of the extract. FME was suspended in distilled water and administered orally to each rat with a volume of 1 ml/100 g body weight.

### 2.2. Isolation and Determination of the Active Compound

**Figure 1:**
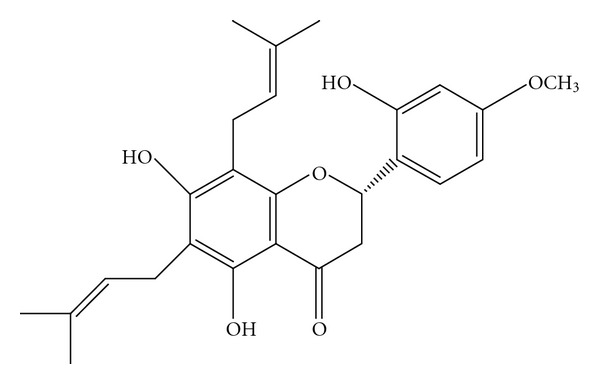
Structure of lespedezaflavanone A.

FME was suspended in water and partitioned with *n*-butanol. The *n*-butanol fraction (FMB) was concentrated, yielding 2.8%. FMB (75 g) was suspended in water and partitioned with chloroform. The chloroform fraction (yield, 21.6 g) was chromatographed on a silica gel (Si 60 F245; Merck, Germany) using hexane/ethyl acetate (80 : 20–0 : 100) to obtain 10 fractions (I–X). Fraction IV (6.1 g) was rechromatographed on a silica gel ODS-18 column (LiChroprep RP-18; Merck) and eluted with methanol/H_2_O (1 : 1–9 : 1) to obtain eight subfractions (a–h). Fraction IV-c (370 mg) was applied to preparative high-performance liquid chromatography (HPLC) to yield a pure compound (75 mg). The conditions for preparative HPLC were as follows: pump, Shimadzu LC-8A (Kyoto, Japan); mobile phase, hexane/ethyl acetate (9 : 1); column, PEGAAIL Silica 60-5 (i.d. 10 mm, 250-mm long; Senshu Scientific, Tokyo, Japan). The pure compound was identified using mass spectroscopy (HP 5995C; USA). Extensive nuclear magnetic resonance analyses (^1^H, ^13^C, DEPT, COXY, HMQC, HMBC; Bruker 400 MHz, Germany) identified the compound as lespedezaflavanone A (LDF-A) [5, 7, 2′-trihydroxy-6, 8-di-*r*, *r*-dimethylallyl-4′-methoxyflavanone] (Figure 1), which had been previously isolated by Wang and Li [9]. ^13^C and ^1^H NMR spectra of LDF-A were as follows: ^1^H NMR (acetone-*d*_6_): *δ* 1.61 (3H, s, H-4′′), *δ* 1.62 (3H, s, H-5′′), *δ* 1.65 (3H, s, H-4′′′), *δ* 1.75 (3H, s, H-5′′′), *δ* 2.79 (1H, dd, *J* = 17.1, 2.9 Hz, H-3), *δ* 3.09 (1H, dd, *J* = 16.8, 12.9 Hz, H-3), *δ* 3.31 (2H, d, *J* = 7.4 Hz, H-1′′), *δ* 3.33 (2H, d, *J* = 7.1 Hz, H-1′′′), *δ* 3.76 (3H, s), *δ* 5.17 (1H, m, H-2′′), *δ* 5.19 (1H, m, H-2′′′), *δ* 5.68 (1H, dd, *J* = 12.8, 2.9 Hz, H-2), *δ* 6.52 (1H, d, *J* = 2.1 Hz, H-3′), *δ* 6.52 (1H, dd, *J* = 9.3, 2.4 Hz, H-5′), *δ* 7.43 (1H, d, *J* = 6.8 Hz, H-6′).^13^C NMR (acetone-*d*_6_): *δ* 17.07 (C-4′′, C-4′′′), 20.06 (C-1′′), 20.94 (C-1′′′), 25.01 (C-5′′, C-5′′′), 41.86 (C-3), 54.63 (C-4′-OCH_3_), 74.35 (C-2), 101.48 (C-5′), 102.44 (C-10), 105.00 (C-3′), 107.11 (C-6), 107.80 (C-8),118.24 (C-1′), 122.51 (C-2′′′), 122.65 (C-2′′), 127.73 (C-6′), 131.07 (C-3′′′), 131.28 (C-3′′), 155.31 (C-2′),158.44 (C-4′), 159.33 (C-7), 160.85 (C-5), 161.42 (C-9), and 197.42 (C-4).

LDF-A content was determined by HPLC. The conditions for HPLC were as follows: pump, Shimadzu LC-6A; UV spectrophotometric detector, SPD-6AV; and column, Cosmosil 5C18-AR-II column (i.d. 4.6 mm, 250-mm long; Nacalai Tesque, Tokyo, Japan). The mobile phase consisted of two elutions programed as a linear gradient of water with 0.2% acetic acid (A) and acetonitrile (B) at a flow rate of 0.3 ml min^−1^ (*t* = 0 minutes, 30% A; *t* = 17 minutes, 20% A; *t* = 40 minutes, 20% A, and next injection). UV detection was performed at 270 nm. LDF-A content in FME was 0.26%.

### 2.3. Mature Osteoclast Differentiation Assay

Bone marrow cells were prepared by removing bone marrow from the femora and tibiae of Wistar rats weighing 250–300 g and then flushing the bone marrow cavity with *α*-minimum essential medium (Hyclone, Logan, UT, USA) supplemented with 20 mM HEPES, 10% heat-inactivated fetal bovine serum, 2 mM glutamine, penicillin (100 U ml^−1^), and streptomycin (100 *μ*g ml^−1^). The nonadherent cells (hematopoietic cells) were collected after 24 hours and used as osteoclast precursors. Cells were seeded at 1 × 10^6^ cells/well in 24-well plates in the presence of human recombinant soluble RANKL (50 ng ml^−1^; PeproTech EC, London, UK) and M-CSF (20 ng ml^−1^; PeproTech EC). Various concentrations of FME or LDF-A were added to these cultures. The culture medium was replaced with fresh medium every 3 days. Osteoclast formation was measured using the tartrate-resistant acid phosphatase (TRAP) staining kit on day 9 [10]. Briefly, adherent cells were fixed with 10% formaldehyde in phosphate-buffered saline (PBS) for 3 minutes and then stained with Naphthol AS-Mx phosphate and tartrate solution for 1 hours at 37°C. TRAP-positive cells with more than three nuclei were scored as osteoclasts [11]. To clarify the action of LDF-A, effect of cotreatment with ICI 182 780 (1 nM), an estrogen receptor (ER) antagonist [12], on the osteoclastogenesis was also investigated.

### 2.4. Animals

Wistar rats were obtained from BioLASCO (Taipei, Taiwan) and housed in an air-conditioned room at 21–24°C with 12 hours of light. The rats had free access to food pellets and water throughout the study period. All the animal experiments were conducted according to the guidelines established by the Animal Care and Use Committee of China Medical University and were approved by this committee.

### 2.5. Animal Experiments

Female rats were anesthetized with pentobarbital sodium (40 mg kg^−1^, i.p.), and their ovaries were removed bilaterally. The rats in the sham-operated group were anesthetized, laparotomized, and sutured without removing their ovaries. After 1 week of recovery from surgery, the OVX rats were randomly divided into five groups and orally treated with H_2_O, FME (50, 250 or 500 mg kg^−1^ daily), or alendronate (2.5 mg kg^−1^ daily; Sigma-Aldrich, St Louis, MO, USA) for 13 weeks. The sham-operated group was orally treated with H_2_O. The body weight of each animal was measured once a week until the final day of administration.

Sixteen-hour urine samples were collected after 13 weeks of treatment. On the last day of the study, the animals were sacrificed under deep anesthesia with a high dose of pentobarbital sodium (65 mg kg^−1^, i.p.), and blood samples were collected simultaneously. The tibiae and lumbar vertebrae were dissected. Each left tibia was submerged in 4% neutral-buffered paraformaldehyde solution and processed to prepare sections for pathological examination. The other dissected bones were stored at −80°C until examination. The vaginae were dissected out and immediately weighed.

### 2.6. Biochemistry

Serum alkaline phosphatase and urinary creatinine were assayed using clinical test kits (Roche Diagnostics, Mannheim, Germany) in a spectrophotometric analyzer (Cobas Mira Plus; Roche, Rotkreuz, Switzerland). Serum calcium (Ca) and urinary Ca were measured by the *o*-cresolphthalein complexone method using a commercial kit (Randox, UK). Urinary Ca contents were expressed as milligrams per millimole of urinary creatinine. Urinary deoxypyridinoline (DPD) contents were determined by enzyme immunoassays using a commercial kit (Metra, San Diego, CA, USA). DPD contents in urine samples were expressed as milligrams per millimole of urinary creatinine.

### 2.7. Measurement of Bone Mineral Content (BMC) and Bone Mineral Density (BMD)

The BMC and BMD of each right tibia were measured using a dual-energy X-ray absorptiometer (XR-26; Norland, Fort Atkinson, WI, USA) using a model for small subjects.

### 2.8. Measurement of Bone Ash and Ca Content

Each fifth lumbar vertebra was desiccated in different baths of alcohol and dried overnight at 100°C. The dry weight was then determined. Next, the vertebra was incinerated for 12 hours at 1000°C, and the ash weight was determined. The measurements obtained were expressed as the percentages of the ash amounts relative to the dry weight of the vertebra. The ash was then solubilized in 6 N HCl and analyzed using a Ca assay. Values were expressed as milligram of Ca per cubic centimeter of bone volume. Bone volume was measured by Archimedes principle [13].

### 2.9. Histological Studies of the Tibiae

The left tibiae were removed, fixed with 4% neutral-buffered paraformaldehyde in PBS (pH 7.4) for 48 hours, and decalcified in 10% ethylenediamine tetraacetic acid solution (pH 7.4) at 4°C for 4 weeks. After decalcification, each bone sample was cut along the coronal plane, embedded in paraffin, and cut longitudinally into sections for histological staining. After staining with hematoxylin and eosin, sections were observed for microarchitectural changes under a microscope. The captured images were analyzed using a color image analysis system (Image-Pro Plus version 5.1; Media Cybernetics, MD, USA). The trabecular bone volume and cortical thickness of the metaphysis were measured. The trabecular bone volume was expressed as a percentage of the total tissue area at the sampling site [14]. For measurement of the osteoclast number, sections were stained for TRAP with TRAP kit (Sigma-Aldrich, St. Louis, MO, USA) as previously described [10].

### 2.10. Statistical Analysis

Results were expressed as mean ± SD. All experimental data were analyzed using one-way analysis of variance with Dunnett's test. Values of *P* < .05 were considered statistically significant.

## 3. Results

### 3.1. Osteoclast Formation from Rat Bone Marrow Cells

**Figure 2:**
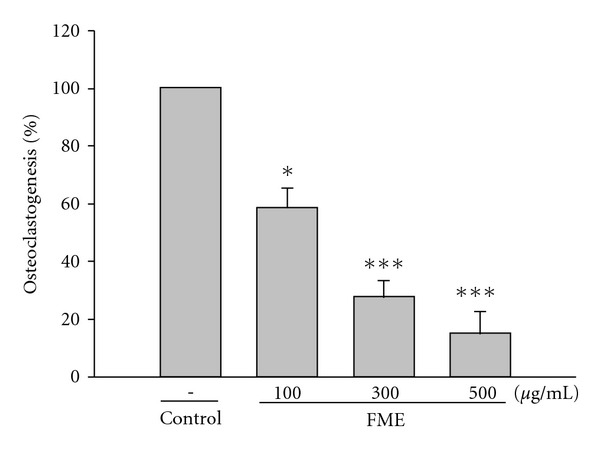
Effects of FME on osteoclastogenesis in rat bone marrow culture system. All values are mean ± SD (*n* = 3). *P < .05, **P < .01, ***P < .001 compared with the control group.

**Figure 3:**
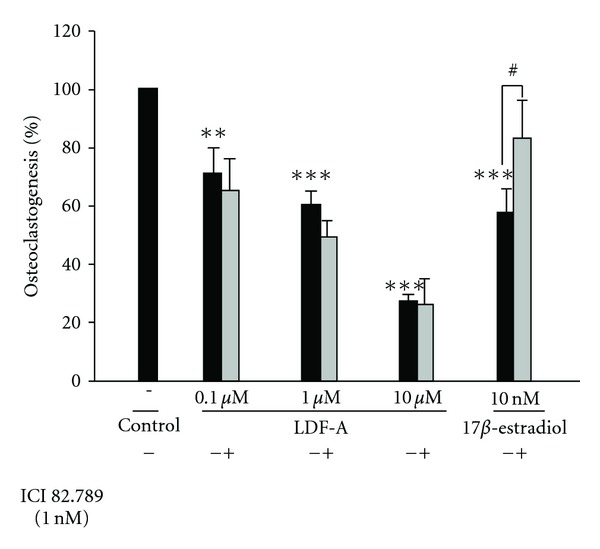
Effect of LDF-A alone and cotreatment with ICI 182 780 (a 17*β*-estradiol antagonist) on osteoclastogenesis in a rat bone marrow culture system. All values are mean ± SD (*n* = 3). **P < .01, ***P < .001 compared with the control group. ^#^ *P* < .05 compared with 17*β*-estradiol group by *t*-test.

The formation of large TRAP-positive multinucleated osteoclasts in bone marrow-derived stromal cell cultures in the presence of M-CSF and RANKL was dose-dependently inhibited by FME (100–500 *μ*g ml^−1^) and LDF-A (10^−7^ to 10^−5^ M) (Figures 2 and 3). In addition, 17*β*-estradiol (10 nM), a positive control drug, showed an inhibitory effect on osteoclastogenesis of rat bone marrow cells (Figure 3). Cotreatment with ICI 182 780 at 1 nM significantly blocked the inhibition of osteoclastogenesis induced by 17*β*-estradiol (Figure 3). However, ICI 182 780 did not reverse these actions of LDF-A (Figure 3). ICI 182 780 alone at 1 nM inhibited osteoclastogenesis by 4%.

### 3.2. Body and Organ Weights

The rats in all of the six experimental groups had similar initial body weights. Thirteen weeks after the operation, the OVX rats showed significant increases in body weight. The increased body weight of the OVX rats was not affected by FME or alendronate administration (Table 1).

The vaginal weights significantly decreased in the OVX rats compared with the sham-operated rats. Decreased vaginal weights in the OVX rats were not affected by FME or alendronate administration (Table 1).

### 3.3. Plasma Alkaline Phosphatase Activity and Urinary Ca and DPD Contents

Ovariectomy induced an increase in the plasma alkaline phosphatase activity. Treatment with FME or alendronate did not affect the plasma alkaline phosphatase activity (Table 2). 

Ovariectomy resulted in a significant increase in urinary Ca and DPD contents in the OVX rats. FME (250 and 500 mg kg^−1^) or alendronate administration suppressed the increase in urinary Ca and DPD contents in OVX rats (Table 2).

### 3.4. BMC and BMD

**Figure 4:**
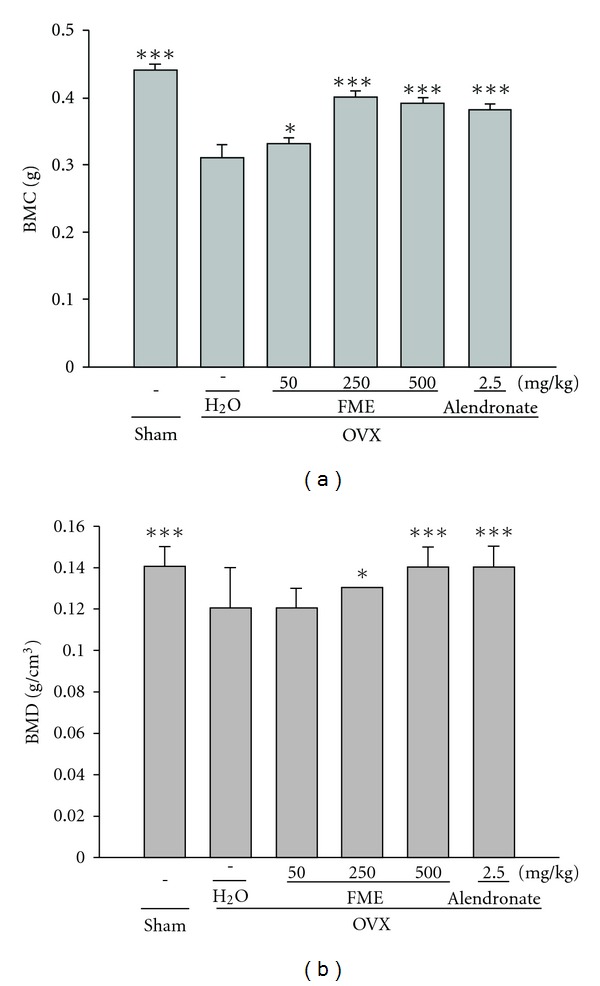
Effects of FME on the BMC and BMD of the right tibia in OVX rats. All values are mean ± SD (*n* = 8). *P < .05, ***P < .001 compared with the OVX + H_2_O group.

The tibial BMC and BMD were 29.5 and 14.3% lower in the OVX group than in the sham group, respectively. Treatment with FME (250 and 500 mg kg^−1^) or alendronate significantly prevented reductions in BMC and BMD (Figure 4).

### 3.5. Bone Ash Ratio and Bone Ca Content

**Figure 5:**
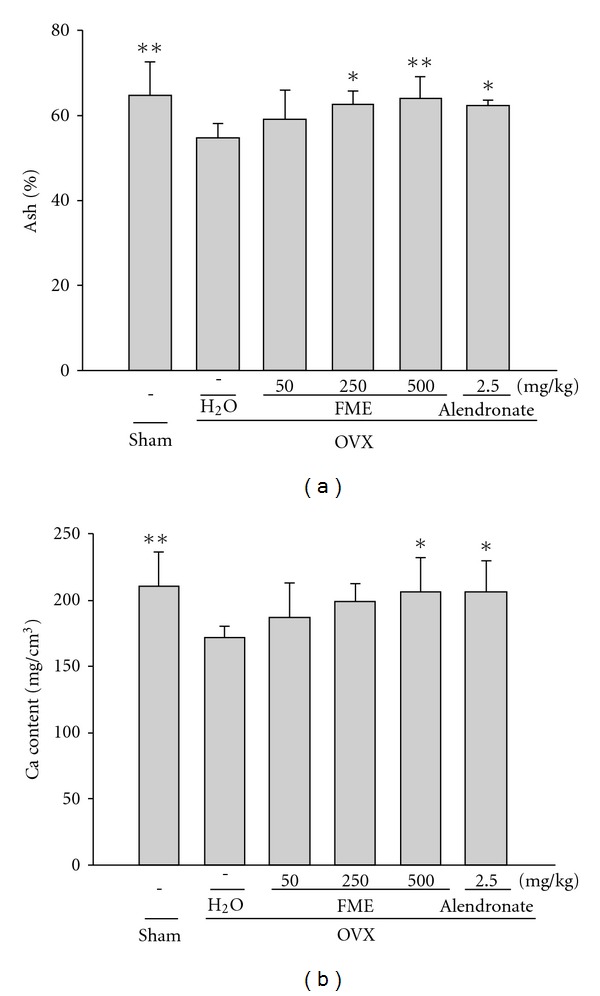
Effects of FME on the ash weight ratio and Ca content of the fifth lumbar vertebra in OVX rats. All values are mean ± SD (*n* = 8). *P < .05, **P < .01, compared with the OVX + H_2_O group.

The ash weight ratio and Ca content of the fifth lumbar vertebra were 15.7 and 18.1% lower in the OVX group than in the control group, respectively. Administration of FME (250 and 500 mg kg^−1^) or alendronate caused significant increase in the ash weight ratio and Ca content of the fifth lumbar vertebra compared with the OVX control group (Figure 5).

### 3.6. Bone Histology

**Figure 6:**
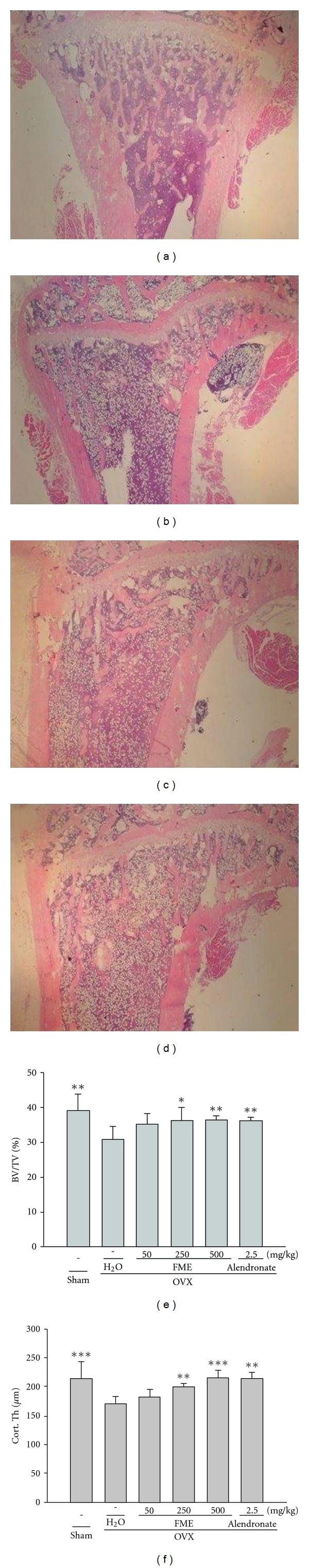
Histological analysis of the bone section on left tibiae. (a) Sham-operated group. (b) OVX + H_2_O group. These rats show decreases in the trabecular bone and cortical thickness of the metaphysis. (c) OVX + FME (500 mg kg^−1^) group. These rats show increases in the trabecular bone and cortical thickness of the metaphysis. (d) OVX + alendronate group. These rats show increases in the trabecular bone and cortical thickness of the metaphysis. Magnification = 40x. (e) Histogram representing image-quantization of the mean percentage of trabecular volume/tissue volume (BV/TV) and (f) cortical thickness (Cort. Th) of the metaphysic. All values are mean ± SD (*n* = 8). *P < .05, **P < .01, ***P < .001 compared with the OVX + H_2_O group.

The trabecular bone volume and cortical thickness of the metaphysis were considerably lower in the OVX rats than in the sham rats. FME and alendronate treatments significantly increased the trabecular bone volume and cortical thickness of the metaphysis in the OVX rats (Figure 6).

**Figure 7:**
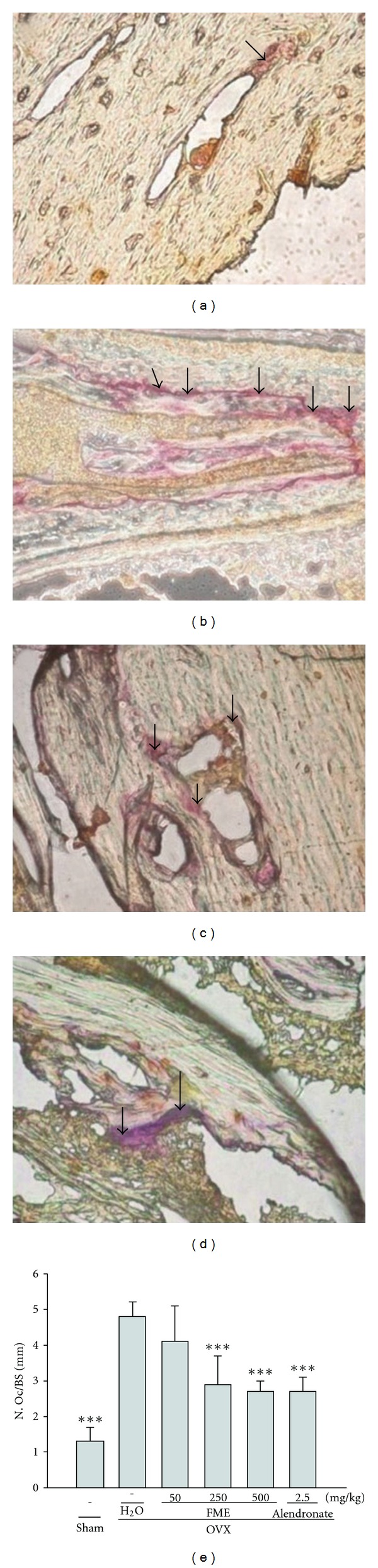
Histological analysis of the number of osteoclasts on the left tibiae. (a) Sham-operated group. (b) OVX + H_2_O group. These rats show an increase in the number of osteoclasts (arrows). (c) OVX + FME (500 mg kg^−1^) group. These rats show a decrease in the number of osteoclasts (arrows). (d) OVX + alendronate group. These rats show a decrease in the number of osteoclasts (arrows). Magnification = 200x. (e) Histogram representing the osteoclast number/mm bone surface (N. Oc/BS). All values are mean ± SD (*n* = 8). ***P < .001 compared with the OVX + H_2_O group.

The number of osteoclasts in the region of the primary spongiosa significantly increased in the OVX rats. FME and alendronate treatments decreased the number of osteoclasts in the OVX rats (Figure 7).

## 4. Discussion

The study has shown that orally administered FME is able to prevent osteoporosis in OVX rats. In addition, its active component, LDF-A, was isolated from FME and found to inhibit osteoclastogenesis *in vitro*, suggesting that it may be useful as a standard compound for FME preparation.

Osteoclasts are generated from hematopoietic cells of the monocyte/macrophage lineage [5]. Several lines of evidence have indicated that two key molecules, M-CSF and RANKL, are essential and sufficient to promote osteoclastogenesis [11]. We used this osteoclast differentiation system to examine the actions of FME and found that it suppressed osteoclast formation. On the basis of the bioactivity-guided fractionation principle, we further isolated the active component, LDF-A, from FME. LDF-A suppressed the formation of osteoclasts in cultured rat bone marrow cells in a dose-dependent manner. LDF-A could be used as a marker component for antiosteoporosis therapies involving FME. We also determined that the amount of LDF-A in FME was 0.26%.

Phytoestrogens are a group of biologically active plant substances with a chemical structure similar to that of estradiol, an endogenous estrogen. This structural similarity accounts for the ability of these compounds to bind to ERs and exert various estrogenic or antiestrogenic effects [15]. There are three main classes of phytoestrogens: isoflavones, coumestans, and lignans, which occur in either plants or seeds [15]. Kuiper et al. [16] reported that the binding affinity of naringenin (flavanone) for ER*β* was 0.11, whereas that of genistein (isoflavone) and kaemferol (flavonol) was 87 and 3, respectively, when the binding affinity for estradiol was arbitrarily set at 100. The affinity of LDF-A may be low, because LDF-A belongs to the same group (flavanone) as naringenin. In this study, we also observed that the inhibitory action of LDF-A on osteoclastogenesis was not abolished by ICI 182 780, an ER antagonist [12], indicating that the inhibitory action of LDF-A on osteoclastogenesis was not dependent on the ER.

To evaluate the efficacy of FME for the treatment of postmenopausal osteoporosis, a rat OVX model was used in this study. Because of the ovarian hormone deficiency, there was an increase in body weight and a decrease in vaginal weight in the OVX rats, consistent with a previous study [17]. ER exists in two main forms, ER*α* and ER*β*, which have distinct tissue expression patterns in both human and rodents [18]. Evidence shows that the selective activation of ER*α* decrease food intake and body weight, body fat, and increase vaginal weight and blood flow in OVX animals [19–21]. FME did not treat the postmenopause associated with obesity and vaginal atrophy, indicating that FME did not exert ER*α* agonist activity.

The OVX model has been used as an animal model for stimulating various clinical syndromes derived from osteoporosis [8]. In this study, histological examination further revealed that the trabecular bone volume and cortical thickness of the metaphysis of the tibia decreased in the OVX rats. In addition, bone loss was manifested by reductions in the tibial BMC and BMD in the OVX rats. Treatment with FME significantly reduced this bone loss in the OVX rats. Bone mineral components, such as Ca, decreased in the fifth lumbar vertebra of the OVX rats, whereas urinary excretion of Ca was elevated in the OVX rats [22]. These results confirm that Ca deposition decreased in OVX rats. FME treatment also improved the loss of Ca content in the OVX rats. These results clearly show that FME treatment in rats can ameliorate osteopenia induced by ovariectomy.

The serum activity of alkaline phosphatase, an index of bone formation [23], has been reported to be significantly greater in an OVX group than in a sham-operated group [24]. A similar change was observed in this study. FME treatment did not affect the activity of serum alkaline phosphatase. DPD is a marker of bone resorption [25]. OVX increased the urinary DPD content, which decreased by FME treatment. These results suggest that FME ameliorated the bone loss induced by ovariectomy by inhibiting bone resorption, rather than enhancing bone formation.

**Figure 8:**
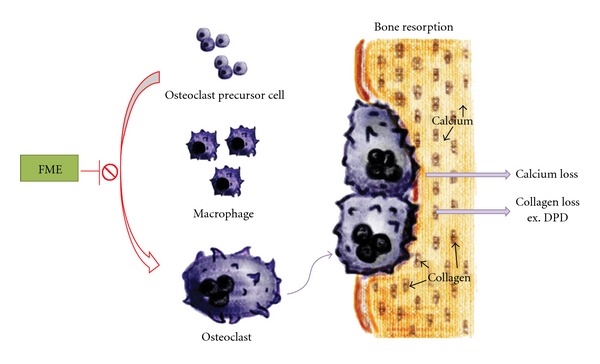
The proposed antiosteoporotic mechanism for FME. FME inhibits the differentiation of bone marrow monocytes to osteoclasts to ameliorate the osteoporosis induced by ovariectomy in mice.

Osteoclasts are multinucleated cells of hematopoietic origin and comprise primary bone-resorbing cells [11]. TRAP is a different form of the enzyme acid phosphatase, which is found mainly in bone and some blood cells. TRAP is released during the bone-resorbing activity of osteoclasts [26]. For measurement of the osteoclast numbers, histological sections were stained with TRAP. We counted the numbers of osteoclasts in the region of the primary spongiosa and found that the numbers decreased in the FME-treated OVX rats. These findings support the idea that FME can ameliorate ovariectomy-induced osteopenia by suppressing osteoclast differentiation (Figure 8).

Both ER receptors are expressed in osteoblasts, octeoclasts, and bone marrow stoma cells, indicating that both ER receptors play a role in skeletal maturation and determination of bone mass [21]. Thus, it is possible that FME ameliorate the osteoporosis in OVX rats through the activation of both ER. Development of tissue-specific selective ER modulators (SERM), such as those specific to the skeleton, might alleviate some of the risk associated with traditional estrogen therapy complicated by effects in other tissues. FME can maintain bone mass, but not effect on obesity and atrophy of vagina, indicating that FME exhibits SERM-like effects.

As a result of evidence that estrogen replacement or bisphosphonates therapies are associated with increased risk of cancer or osteonecrosis, respectively [27, 28]. It is now generally recognized that alternative approaches to the prevention and treatment of osteoporosis might be worth exploring. Scientists have turned to explore the health benefits of plant-based bioactive compounds that have been shown to positively influence bone metabolism. For instance, soy-based diets plus fructooligosaccharides, and *Epimedium brevicornum*, a Chinese herbal medicine, have been shown to improve bone mass in animal models of osteoporosis [22, 29]. Therefore, we undertook this study to evaluate the efficacy of FME, a Taiwanese herb, in preventing osteoporosis using the OVX model. Our study had shown that FME exhibits antiosteoporotic effects, suggesting that it may play a promising role in the treatment of osteoporosis.

## Funding

This study was supported by grants from the China Medical University (CMU 96-076).

## Figures and Tables

**Table 1:** Effects of FME on body weight and weight of vigina in OVX rats.

Drugs	Dose (mg kg^−1^)	Body weight (g)		Vaginal (g)
		Week 0	Week 13	
Sham	—	267.1 ± 13.6	289.6 ± 19.0***	0.50 ± 0.04***
OVX + H_2_O	—	260.0 ± 22.7	372.8 ± 30.7	0.17 ± 0.02
OVX + FME	50	263.7 ± 18.4	378.8 ± 34.2	0.18 ± 0.02
	250	263.8 ± 23.0	369.1 ± 27.6	0.17 ± 0.03
	500	260.3 ± 18.0	374.8 ± 25.7	0.17 ± 0.02
OVX + Alen	2.5	266.6 ± 20.1	377.8 ± 36.5	0.18 ± 0.01

All values are mean ± SD (*n* = 8). Alen, alendronate.

***P < .001, versus the OVX + H_2_O group.

**Table 2:** Effects of FME on serum alkaline phosphatase activity and urinary Ca and DPD contents in OVX rats.

Drugs	Dose	ALP	Urinary Ca	Urinary DPD
	(mg kg^−1^)	(IU l^−1^)	(mg mmol^−1^ Crea.)	(nmol mmol^−1^ Crea.)
Sham	—	120.6 ± 24.6**	39.7 ± 21.0**	292.4 ± 58.7***
OVX + H_2_O	—	167.1 ± 25.0	89.3 ± 40.3	520.7 ± 97.0
OVX + FME	50	160.9 ± 38.5	52.5 ± 12.4	444.7 ± 104.2
	250	156.3 ± 12.5	39.3 ± 20.3*	384.5 ± 106.2*
	500	150.6 ± 20.0	37.3 ± 15.3**	380.4 ± 69.5*
OVX + Alen	2.5	163.5 ± 24.0	45.2 ± 14.8**	281.2 ± 52.3***

All values are mean ± SD (*n* = 8). Alen, alendronate; ALP, alkaline phosphatase; Crea., creatinine.

*P < .05, **P < .01, ***P < .001, versus the OVX + H_2_O group.
